# The prevalence and associated factors of compassion fatigue among pediatric intensive care unit nurses in Southwest China: a cross-sectional study

**DOI:** 10.3389/fpubh.2026.1879953

**Published:** 2026-07-07

**Authors:** Zhangqin Liu, Shuai Zhang, Lei Lei, Cheng Yang, Min Xu

**Affiliations:** 1 Department of Pediatric Intensive Care Unit Nursing, West China Second University Hospital, Sichuan University, Chengdu, Sichuan, China; 2 Key Laboratory of Birth Defects and Related Diseases of Women and Children (Sichuan University), Ministry of Education, Chengdu, Sichuan, China

**Keywords:** associated factors, compassion fatigue, intensive care units, pediatric, nurses, surveys and questionnaires

## Abstract

**Background:**

Compassion fatigue, a form of emotional exhaustion resulting from repeated exposure to patients’ suffering and trauma, is prevalent among nurses in pediatric intensive care units (PICUs). It damages nurses’ health and reduces care safety and quality. This study aims to investigate the prevalence and associated factors of compassion fatigue among PICU nurses in China.

**Methods:**

A cross-sectional study was conducted among 186 PICU nurses from seven hospitals in Chengdu, China, using convenience sampling. Data were collected using the Professional Quality of Life Scale (Pro-QOL) and the Social Support Rating Scale, along with assessments of sociodemographic characteristics, work-related information, and self-perceived health status. According to the Pro-QOL Manual, compassion fatigue comprises two distinct subdimensions: burnout and secondary traumatic stress.

**Results:**

The study found that 74.7% of PICU nurses reported moderate to high secondary traumatic stress, and 79.6% reported moderate to high burnout. In contrast, 24.7% reported high compassion satisfaction. Multiple linear regression identified several significant factors associated with the subdimensions of professional quality of life. Higher social support was significantly associated with higher compassion satisfaction (*β* = 0.375, *p* < 0.001) and lower burnout (*β* = −0.224, *p* < 0.001). Poorer self-perceived health, worse sleep quality, more frequent end-of-life care experiences, and less frequent gratitude from patients/families were also significantly linked to higher burnout or secondary traumatic stress (all *p* < 0.05).

**Conclusion:**

The high prevalence of burnout and secondary traumatic stress among PICU nurses underscores the need for targeted interventions. Interventions should prioritize strengthening social support systems, improving sleep quality, and providing additional support for nurses frequently engaged in end-of-life care.

## 1 Background

Compassion fatigue (CF), a form of emotional exhaustion, is prevalent among healthcare workers, particularly nurses, due to repeated exposure to patients’ suffering and trauma. It manifests as emotional numbness, burnout, and secondary traumatic stress (STS) (1), and is often described as the “cost of caring” (2). According to Coetzee and Klopper, CF occurs when nurses expend more empathetic energy than they can replenish, leading to a diminished capacity for recovery. The concept of “professional quality of life” provides a comprehensive framework for understanding the caregiver experience, which encompasses both the positive rewards (compassion satisfaction) and the negative costs, notably compassion fatigue comprised of burnout and secondary traumatic stress (2–4).

As the largest group of healthcare providers, nurses are uniquely responsible for addressing patients’ physical, mental, emotional, and spiritual needs—a role that other healthcare professionals rarely undertake (5–7). International studies report that the incidence of CF among nurses ranges from 21.6 to 44.8% (8–10), while in China, the rate of moderate to high levels of CF exceeds 80% (11, 12). This disparity may be attributed to differences in hospital regions and departmental categories. This condition not only affects nurses’ physical and mental well-being but may also result in reduced nursing care quality, lower patient satisfaction, and an elevated risk of medical errors (13, 14).

Pediatric Intensive Care Unit (PICU) nurses face unique challenges that further increase their vulnerability to CF. In the PICU, nurses care for critically ill children and must simultaneously address the heightened emotional needs of their families. Prolonged exposure to such high-stress environments, combined with frequent encounters with life-and-death situations, places PICU nurses at a significantly higher risk of CF compared to nurses in other departments (15). The COVID-19 pandemic has further exacerbated this issue, as healthcare workers globally have faced unprecedented emotional and psychological burdens (16).

A meta-analysis involving 28,509 nurses reported that high levels of compassion fatigue have multiple detrimental effects. At the nurse level, it leads to insomnia, depression, and reduced professional accomplishment; at the patient level, it results in strained nurse–patient relationships, decreased quality of care, and increased medical disputes; at the hospital level, it contributes to staff turnover, increased operational costs, and reduced work efficiency (17). Research has identified several key factors that influence the development of CF, including work environment, social support, workload, and quality of life (18). In the PICU, nurses face distinct stressors arising from the vulnerability of critically ill children and the heightened emotional demands of their families, which collectively elevate CF risk, a situation worsened by inadequate social and psychological resources (19, 20). While these general factors are recognized, their interplay within the unique PICU context—marked by a “dual” emotional burden—remains underexplored. Although CF in ICU settings has garnered attention, studies specifically targeting the PICU environment are scarce. In China, existing research has predominantly examined adult ICUs, emergency departments, or general nursing populations, thereby neglecting the particular stress ecosystem inherent to PICU care (21, 22).

Given that PICU nurses serve as critical guardians of children’s lives, investigating the prevalence of compassion fatigue in this population and elucidating its associated factors is essential for developing effective interventions and support systems. Therefore, this study aims to examine the prevalence of CF among PICU nurses in Southwest China and its associations with work-related factors and social support.

## 2 Methods

### 2.1 Study setting and sample

A cross-sectional survey was conducted across seven hospitals in Chengdu, a city in southwestern China. A convenience sampling method was used, and an online survey was administered to nurses working in PICUs in March 2023. Inclusion Criteria: (1) hold a current nursing practice qualification; (2) currently working in PICU, with a work duration of ≥3 months; (3) informed consent and voluntary participation. Exclusion Criteria: (1) nursing staff on further education, rotation, internship, standardized training, or on leave for more than 2 weeks during the survey period; (2) nursing staff with a confirmed diagnosis of mental disorders, such as anxiety disorders, depression, etc.; (3) refusal to cooperate.

The sample size was determined based on the recommended minimum of 10 observations per predictor variable for multiple linear regression (23). With up to 15 predictor variables planned for inclusion in the final model, a minimum of 150 valid observations was required. To account for potential incomplete responses and attrition, and assuming a questionnaire response rate of 80%, we aimed to recruit at least 188 participants. The final sample of 186 valid responses met this requirement.

### 2.2 Data collection instruments

#### 2.2.1 Sociodemographic characteristics

The study considered various sociodemographic factors, including gender, age, marital status, educational attainment, and monthly income, expressed in Chinese yuan (CNY; where 1 CNY is approximately equal to 0.15 USD).

#### 2.2.2 Work-related information

The study considered various work-related information, including years of working in the hospital (<1 year, 1–2 years, 3–5 years, 6–10 years, and ≥11 years); professional title (nurse, junior nurse, senior nurse, associate chief nurse, chief nurse); position type (administration, clinical, teaching, research); number of children cared for during day shift (1, 2, 3, 4, 5 or more); number of children cared for during night shift (2, 3, 4, 5, 6 or more); frequency of emergency resuscitations experienced (once per shift, once per week, once every 2 weeks, once per month, once per year, no experience); frequency of end-of-life care experienced (once per shift, once per week, once every 2 weeks, once per month, once per year, no experience); frequency of family thanks (10 or more times per year, 6–9 times per year, 3–5 times per year, 1–2 times per year, No experience).

#### 2.2.3 The other related information

The other related information including self-perceived health status (good, average, not good); sleep states (very good, good, poor, very poor).

#### 2.2.4 Professional Quality of Life Scale (Chinese version)

The Professional Quality of Life Scale (Pro-QOL), developed by Stamm (24). encompasses three key dimensions: compassion satisfaction (CS), secondary traumatic stress (STS), and burnout (BO). According to the Pro-QOL Manual, compassion fatigue (CF) comprises two distinct subdimensions: burnout and secondary traumatic stress, while compassion satisfaction (CS) represents the positive aspect of professional quality of life. This scale has gained extensive validation and is employed to assess professional quality of life within a diverse array of helping professions. For the purposes of this study, the Chinese adaptation of the scale, translated and proofread by H Chen (25), was utilized, as it is particularly well-suited to the Chinese nursing population. The scale as a whole boasts a Cronbach’s alpha coefficient of 0.91, a split-half reliability coefficient of 0.86, and individual Cronbach’s alpha coefficients for its internal dimensions of 0.88, 0.75, and 0.81, demonstrating robust reliability and validity. In the current sample, the Cronbach’s alpha coefficient for the total Pro-QOL scale was 0.847, indicating good internal consistency. Comprising 30 items across its three dimensions, the scale employs a 5-point Likert scoring system, with responses ranging from “Never” to “Always,” corresponding to scores from 1 to 5, respectively.

According to the Pro-QOL Manual, the scale is best used in its continuous form. However, to indicate relative risks or protective factors, cut scores are provided at the 25th and 75th percentiles. For compassion satisfaction, the 25th and 75th percentiles are 44 and 57, respectively; for burnout, they are 43 and 56; and for secondary traumatic stress, they are 42 and 56. Scores below the 25th percentile indicate low levels, scores between the 25th and 75th percentiles indicate moderate levels, and scores above the 75th percentile indicate high levels. This study adopted the categorization method from the Pro-QOL Manual to classify the results of the present study. The Pro-QOL is intended for screening and planning purposes only and is not a diagnostic tool.

#### 2.2.5 Social Support Rating Scale

The Social Support Scale, compiled by Chinese scholar S Xiao, has been widely applied in China. The Cronbach’s alpha coefficients for each dimension range from 0.89 to 0.94, and the test–retest reliability is 0.92, indicating good reliability and validity (26). In the current sample, the Cronbach’s alpha coefficient for the total scale was 0.821, indicating acceptable internal consistency. The scale consists of three dimensions: objective support, subjective support, and the utilization of social support. We used this scale to measure the social support received and utilized by PICU nurses. The total score is the sum of the scores of all 10 items, with each dimension’s score being the sum of the corresponding item scores. The total score ranges from 12 to 66 points, with the following ranges for each dimension: objective support from 1 to 22 points, subjective support from 8 to 32 points, and the utilization of social support from 3 to 12 points. Higher scores on the scale indicate a higher level of social support.

### 2.3 Investigation methods

All the above-mentioned items in the assessment tools were integrated into the same questionnaire and presented in the form of an online questionnaire. The formal survey was conducted using an electronic questionnaire distributed online. The questionnaire link was sent by the researchers to the head nurses of PICUs in various hospitals in Chengdu. The head nurses then distributed the link to the PICU nurses in their respective hospitals. At the top of the questionnaire interface, a unified set of instructions was displayed, outlining the purpose, significance, and process of the study. It also informed participants that the entire survey would be anonymous, that strict confidentiality measures would be applied to the data, and that they could withdraw from the questionnaire at any time.

To prevent duplicate submissions and careless responses, this study implemented the following measures on the survey platform: limiting responses to one submission per IP address, manually reviewing questionnaires completed in an excessively short time (<5 min), and excluding responses showing patterned or non-genuine answering behaviors.

### 2.4 Data analysis

Statistical analysis was conducted using SPSS 25.0 software, with a significance level set at *p* < 0.05. Continuous data were described using mean ± standard deviation or median and interquartile range, while categorical data were described using frequency and composition ratio. For univariate analysis, we employed analysis of variance, t-tests, or non-parametric tests to compare the demographic characteristics, levels of social support, and CF among PICU nurses. Additionally, Spearman correlation coefficient methods were used to analyze the correlation between PICU nurses’ CF and social support.

Prior to multiple linear regression, the following assumptions were tested: normality of residuals (Shapiro–Wilk and P–P plots), homoscedasticity (residual plots), and multicollinearity (VIF). Collinearity diagnostics showed no multicollinearity. For stepwise regression, variables were entered with *p* < 0.05 in univariate and correlation analyses were included as independent variables.

## 3 Results

### 3.1 General information of PICU nurses

A total of 194 questionnaires were distributed. Among them, 5 were not submitted. Of the submitted questionnaires, 2 were excluded because the completion time was <5 min, and 1 were excluded due to patterned answering behaviors. After these exclusions, 186 valid questionnaires were retained for final analysis, which is consistent with the planned sample size of 188.

This survey included a total of 186 participants, predominantly females, accounting for 158(84.9%) people. In terms of age, most participants fell into the “26–35 years” age group, making up 62.9% of the total. Regarding marital status, the distribution was relatively even between married and single individuals, at 51.1 and 47.8%, respectively. In terms of frequency of emergency resuscitations experienced, 33.9% of the participants did so at least once a week, and 24.7% at least once a month. In contrast, the frequency of end-of-life care experienced for children was generally low, with the majority participating at least once a year, accounting for 56.5%. The frequency with which PICU nurses received thanks from children or their families varied, with “3–5 times a year” accounting for 26.3% and “1–2 times a year” accounting for 26.9%; 13.4% of the participants reported never having received thanks. Regarding the self-perceived health status of PICU nurses, those who described their health as “average” or “good” accounted for 98.4%, while those who chose “poor” health made up 1.6%. As for sleep quality in the past month, 44.6% of the nurses reported good sleep quality, while 41.4% reported “poor” sleep quality. Specific details are shown in Table 1.

**Table 1:** Demographic and occupational characteristics of PICU nurses (*N* = 186).

Characteristic	Subcategory	Frequency (*n*)	Composition ratio (%)
Gender	Male	28	15.1
	Female	158	84.90
Age	≤25 years old	34	18.3
	26–35 years old	117	62.9
	36–45 years old	34	18.3
	≥46 years old	1	0.5
Marital status	Single	89	47.8
	Married	95	51.1
	Others	2	1.1
Educational attainment	Junior high school or below	30	16.1
	College/university	153	82.3
	Master’s degree or above	3	1.6
Monthly income (CNY)	3,000 and below	5	2.7
	3,000 ~ 6,000	40	21.5
	7,000 ~ 9,000	70	37.6
	More than 10,000 yuan	71	38.2
Years of work in the hospital	<1 year	13	7.0
	1–2 years	28	15.1
	3–5 years	40	21.5
	6–10 years	60	32.3
	≥11 years	45	24.2
Professional title	Nurse	38	20.4
	Junior nurse	92	49.5
	Senior nurse	52	28.0
	Chief nurse	4	2.2
Number of children cared for during day shift	1	6	3.2
	2	107	57.5
	3	63	33.9
	4	5	2.7
	5 or more	5	2.7
Number of children cared for during night shift	2	27	14.5
	3	74	39.8
	4	67	36.0
	5	8	4.3
	6 or more	10	5.4
Frequency of emergency resuscitations experience	Once per shift	10	5.4
	Once per week	63	33.9
	Once every 2 weeks	38	20.4
	Once per month	46	24.7
	Once per year	24	12.9
	No experience	5	2.7
Frequency of end-of-life care experience	Once per shift	2	1.1
	Once per week	3	1.6
	Once every 2 weeks	13	7.0
	Once per month	47	25.3
	Once per year	105	56.5
	No experience	16	8.6
Frequency of gratitude from the child or their family	≥10 times/year	45	24.2
	6 ~ 9 times/year	17	9.1
	3 ~ 5 times/year	49	26.3
	1–2 times/year	50	26.9
	No experience	25	13.4
Self-perceived health status	Good	120	64.5
	Average	63	33.9
	Not good	3	1.6
Sleep states in the last month	Very good	15	8.1
	Good	83	44.6
	Poor	77	41.4
	Very poor	11	5.9

### 3.2 Scores of the professional quality of life scale (pro-QOL) in PICU nurses

The Shapiro–Wilk normality test indicated that the data for the Compassion Satisfaction and Burnout dimensions met the assumption of normality (*p* > 0.05), while the data for the Secondary Traumatic Stress dimension did not satisfy the normality assumption (*p* < 0.05). The score of compassion satisfaction dimensions was (30.33 ± 7.80), with a minimum value of 12 and a maximum value of 50. The median value of secondary traumatic stress dimensions were 21.00 (17.00, 25.00), with a minimum value of 11 and a maximum value of 34. The score of burnout dimensions was (25.93 ± 4.26), with a minimum of 15 and a maximum of 39. Specific details are shown in Table 2.

**Table 2:** Scores of pro-QOL in PICU nurses (*N* = 186).

Dimension	Minimum	Maximum	^−^X ± S/M(P25, P75)
Compassion satisfaction (CS)	12	50	30.33 ± 7.80
Secondary traumatic stress (STS)	11	34	21.00 (17.00, 25.00)
Burnout (BO)	15	39	25.93 ± 4.26

### 3.3 Distribution of compassion satisfaction, secondary traumatic stress, and burnout levels among PICU nurses

Table 3 presents the distribution of the three Pro-QOL subscales among PICU nurses. According to the Pro-QOL Manual cutoffs (25th and 75th percentiles), 24.7% of nurses reported high compassion satisfaction (≥ 57), while 31.7 and 24.2% reported high secondary traumatic stress (≥ 56) and high burnout (≥ 56), respectively.

**Table 3:** Distribution of compassion satisfaction, secondary traumatic stress, and burnout levels among PICU nurses (*N* = 186).

Dimension	Level	*n*	Composition ratio (%)
Compassion satisfaction (CS)	≤44	54	29.0
	45–56	86	46.2
	≥57	46	24.7
Secondary traumatic stress (STS)	≤42	47	25.3
	43–55	80	43.0
	≥56	59	31.7
Burnout (BO)	≤43	38	20.4
	44–55	103	55.4
	≥56	45	24.2

### 3.4 Scores of social support rating scale in PICU nurses

The Shapiro–Wilk normality test indicated that the total social support score satisfied the assumption of normality (*p* > 0.05), whereas the scores for the objective support dimension, subjective support dimension, and social support utilization dimension did not meet the normality assumption (*p* < 0.05). The total score of social support for PICU nurses was 37.81 ± 8.49 points, with a minimum score of 18.00 points and a maximum score of 60.00 points (Table 4).

**Table 4:** PICU nurses’ scores of social support rating scale and each dimension (*N* = 186).

Dimension	Minimum	Maximum	^−^X ± S/M(P25, P75)
Scores of objective support dimension	1.00	18.00	8.00 (7.00 ~ 11.00)
Scores of subjective support dimension	11.00	32.00	21.00 (17.00 ~ 25.00)
Scores of social support utilization dimension	3.00	12.00	8.00 (6.00 ~ 9.25)
Total scores of social support rating scale	18.00	60.00	37.81 ± 8.49

### 3.5 Factors associated with the CS, STS and BO: univariate analysis

The key results of the univariate analyses are summarized narratively below; detailed statistical values are available from the corresponding author upon request. After normality testing, the scores for the secondary traumatic stress dimension were found to be non-normally distributed; therefore, the Mann–Whitney U or Kruskal–Wallis test was used. The scores for the compassion satisfaction and burnout dimensions both met the assumptions of normality and homogeneity of variance; thus, independent samples t-tests or one-way ANOVA were selected for statistical analysis.

The results showed that there were significant differences in compassion satisfaction scores across individuals self-perceived health status (*F* = 13.987, *p* < 0.001) and sleep states in the last month (*F* = 10.628, *p* < 0.001). Additionally, significant differences were observed in the scores of secondary traumatic stress dimensions, which were influenced by self-perceived health status (*F* = 7.243, *p* = 0.027), the frequency of end-of-life care experience for children (*F* = 15.433, *p* = 0.009), and the frequency of gratitude from the child or their family (*F* = 12.332, *p* = 0.015). Furthermore, the scores related to burnout dimensions showed significant disparities, which were associated with self-perceived health status (*F* = 24.458, *p* < 0.001), the frequency of end-of-life care experience for children (*F* = 2.951, *p* = 0.014), and sleep states in the last month (*F* = 13.84, *p* < 0.001).

### 3.6 Correlation analysis between CF and social support in PICU nurses

Spearman correlation analysis (Table 5) showed that compassion satisfaction (CS) was positively correlated with all social support dimensions and the total score (all *p* < 0.01), while burnout (BO) was negatively correlated with these variables (all *p* < 0.01), and secondary traumatic stress (STS) was negatively correlated with social support utilization (*p* < 0.01). According to Cohen’s guidelines (*r* = 0.10–0.29 = weak, 0.30–0.49 = moderate, ≥0.50 = strong) (27), the CS-total social support correlation (*r* = 0.505) was strong; the BO-social support correlations (*r* = −0.313 to −0.435) were moderate to strong; and the STS-social support utilization correlation (*r* = −0.242) was weak.

**Table 5:** Spearman correlation coefficients between CF dimensions and Social Support scores (*N* = 186).

Variable	CF			Social Support Rating Scale			
	CS	STS	BO	OSD	SSD	SSUD	TSSS
CS	1						
STS	0.008	1					
BO	−0.590^**^	0.535^**^	1				
OSD	0.399^**^	−0.068	−0.313^**^	1			
SSD	0.425^**^	−0.031	−0.255^**^	0.589^**^	1		
SSUD	0.439^**^	−0.242^**^	−0.435^**^	0.335^**^	0.446^**^	1	
TSSS	0.505^**^	−0.105	−0.375^**^	0.799^**^	0.923^**^	0.616^**^	1

^*^p < 0.05; **p < 0.01. OSD, objective support dimension; SSD, subjective support dimension; SSUD, social support utilization dimension; TSSS, total scores of social support rating scale.

### 3.7 A multiple linear regression analysis of CS, STS and BO

Prior to conducting the regression analysis, we coded the categorical variables included in the analysis as follows. Self-perceived health status was coded as 1 = good, 2 = average, 3 = not good. Sleep states in the last month were coded as 1 = very good, 2 = good, 3 = poor, 4 = very poor. Frequency of end-of-life care experience was coded as 1 = once per shift, 2 = once per week, 3 = once every 2 weeks, 4 = once per month, 5 = once per year, 6 = no experience. Frequency of family thanks was coded as 1 = ≥10 times/year, 2 = 6–9 times/year, 3 = 3–5 times/year, 4 = ≤2 times/year, 5 = 0 times. Social support variables were treated as continuous variables based on the original scale scores.

In linear regression analysis, the variables that enter the CS dimension regression equation include total scores of social support rating scale, self-perceived health status, and sleep states in the last month. The variables that enter the regression equation of the STS dimension include the scores of social support utilization, the frequency of end-of-life care experience, and the frequency of gratitude from the child or their family. The variables that enter the BO dimension regression equation include self-perceived health status, the frequency of end-of-life care experience, the scores of social support utilization, and sleep states in the last month. Specific details are shown in Tables 6–8.

**Table 6:** Multiple linear regression analysis of factors associated with compassion satisfaction among PICU nurses (*N* = 186).

Variable	*B*	*β*	*t*	*p*	VIF
(Constant)	30.044	/	8.916	0	/
Total score for social support	0.345	0.375	6.156	<0.001^*^	1.142
Self-perceived health status	−2.416	−0.160	−2.561	0.011^*^	1.203
Sleep states in the last month	−1.451	−0.135	−2.111	0.036^*^	1.268

*R* = 0.643, *R*^2^ = 0.413, Adjusted *R*^2^ = 0.400, *F* = 31.848, ^*^ p < 0.05.

**Table 7:** Multiple linear regression analysis of factors associated with secondary traumatic stress among PICU nurses (*N* = 186).

Variable	*B*	*β*	*t*	*p*	VIF
(Constant)	28.258	/	10.336	0	/
The scores of social support utilization	−0.490	−0.190	−2.676	0.008^*^	1.062
Frequency of end-of-life care experience	−0.931	−0.159	−2.283	0.024^*^	1.016
frequency of family thanks	−0.541	−0.143	−2.055	0.041	1.014

*R* = 0.373, *R*^2^ = 0.139, Adjusted *R*^2^ = 0.120, *F* = 7.309, ^*^ p < 0.05.

**Table 8:** Multiple linear regression analysis of factors associated with burnout among PICU nurses (*N* = 186).

Variable	*B*	*β*	*t*	*p*	VIF
(Constant)	25.490	/	11.708	0	/
Self-perceived health status	2.345	0.270	4.455	<0.001^*^	1.202
The scores of social support utilization	−0.501	−0.224	−3.750	<0.001^*^	1.172
Frequency of end-of-life care experience	−0.839	−0.165	−2.953	0.004^*^	1.023
Sleep states in the last month	1.061	0.172	2.699	0.008^*^	1.329

*R* = 0.672, *R*^2^ = 0.451, Adjusted *R*^2^ = 0.436, *F* = 29.615, ^*^ p < 0.05.

## 4 Discussion

The study found that 74.7% of PICU nurses reported moderate to high secondary traumatic stress, and 79.6% reported moderate to high burnout. This finding is consistent with the research by Panagou et al. (28), who reported that 74.8% of PICU healthcare professionals were at moderate risk for CF. Some studies have shown that a deterioration in general health status is significantly associated with an increase in secondary traumatic stress levels, suggesting that physical health impairment may exacerbate psychological occupational risks (29). The higher levels of CF among PICU nurses in our study may be attributed to the unique challenges they face. Constantly grappling with the high expectations of patient and family care, often without proper remuneration, may lead to neglect of their own well-being and overexertion that exceeds their personal boundaries. Subsequently, this can cause a decline in the level of compassion satisfaction they feel (28, 30). Furthermore, the high-acuity environment, including frequent alarm noises and confined spaces, may contribute to elevated stress and emotional exhaustion (14).

In addition to work-related factors, our findings indicate that nurses with better self-perceived health status and satisfactory sleep quality are less likely to experience CF. This aligns with prior research indicating that poor health status can lead to feelings of helplessness and negative emotions, thereby contributing to CF (31–33). Poor sleep quality has also been linked to increased susceptibility to CF, as chronic sleep deprivation can negatively impact work performance and overall physical and mental health (17). The physically demanding nature of PICU work, including prolonged standing, night shifts, and high cognitive load, can erode nurses’ physiological reserves over time. When combined with inadequate recovery through poor sleep, this depletion may lower their threshold for emotional exhaustion. Therefore, maintaining nurses’ health and sleep quality should be viewed not merely as personal wellbeing issues, but as essential components of occupational safety and care quality. Hospital administrators should consider sleep hygiene programs, reasonable shift scheduling, and regular health check-ups as institutional priorities rather than optional benefits.

This study showed that PICU nurses who more frequently engage in end-of-life care for children tend to have higher scores of CF. The ineffective treatment, worsening condition, and ultimate death of pediatric patients serve as significant stressors for PICU nurses. In our regression analysis, the frequency of end-of-life care was a significant predictor of both STS (*β* = −0.159) and BO (β = −0.165), indicating a meaningful effect. This finding is consistent with previous research showing that witnessing resuscitation, end-of-life issues, and patient deaths in the ICU can trigger traumatic experiences, including helplessness, frustration, and guilt among nurses. Interventions such as cultivating a compassionate organizational culture, providing ongoing professional training on trauma dynamics and self-care, and recognizing the individual contributions of nurses are conducive to nurses’ recovery from the emotional impact of trauma (34). On the other hand, nurses who receive more gratitude from patients or their families are less likely to experience CF. Kelly (35) suggests that meaningful recognition, such as gratitude from patients, can serve as a protective factor against CF. These findings underscore the importance of providing emotional support and recognition to PICU nurses, particularly those frequently involved in end-of-life care.

This study found that PICU nurses with higher levels of social support and those who are more adept at utilizing it have relatively lower levels of CF. Social support significantly enhances compassion satisfaction among PICU nurses, consistent with the findings of Li (36). Early research has indicated that social support has a universally beneficial effect (37), not only helping nurses better manage work–family conflicts but also providing emotional support and serving as a protective factor for mental health (38). Multiple studies have explored the link between social support and CF, highlighting its regulatory potential to mitigate the intensity and direction of CF (39, 40).

This survey revealed that the majority of PICU nurses (71.5%) have a moderate level of social support, suggesting that nursing managers could further leverage this protective factor by organizing activities, implementing incentive strategies (35, 41), and fostering leadership and peer support to enhance nurses’ social support and promote their overall well-being (42, 43). Similarly, Yang found in the context of gynecological recurrent pregnancy loss care that systematic support at the organizational level, including formal debriefing mechanisms, adequate staffing, and specialized training, significantly improved nurses’ emotional regulation and reduced compassion fatigue (44). Further supporting the importance of social support, a study on PICU nurses showed that implementing a cumulative stress debriefing one-on-one support program can alleviate CF, improve nurse retention and job satisfaction, provide a safe space for sharing feelings and building connections, and help nurses develop coping mechanisms (45).

This study has several limitations. First, the sampling was limited to PICU nurses from seven hospitals in Chengdu via convenience sampling, and the sample size was relatively small; thus, the findings may lack generalizability to broader populations. Second, as a cross-sectional design, it cannot establish causality or evaluate interventions. Third, because questionnaires were distributed via PICU head nurses, hierarchical influence or response bias may have been introduced, as some nurses might have been reluctant to disclose true feelings about work-related stress. Future studies should consider more anonymous distribution methods. Fourth, stepwise regression was used for predictor selection, which is a data-driven method that may lead to overfitting. Future studies with larger samples and theory-driven modeling strategies are needed to validate our findings. Finally, although multicollinearity was controlled for, high correlations between some variables may still influence coefficient interpretability. Future studies should consider multi-center designs, longitudinal or mixed methods, and more inclusive sampling to address these limitations.

## 5 Conclusion

This study reveals a high prevalence of burnout and secondary traumatic stress among PICU nurses in China, with 79.6% reporting moderate-to-high burnout and 74.7% reporting moderate-to-high secondary traumatic stress. Key factors associated with CF include self-perceived health, sleep quality, end-of-life care experiences, and social support. Higher social support was associated with lower CF, highlighting its protective role. These findings may inform targeted interventions, including enhancing social support, improving sleep quality and self-perceived health, and providing additional support for those frequently involved in end-of-life care.

## Data Availability

The raw data supporting the conclusions of this article will be made available by the authors, without undue reservation.
